# Supplementary material for: Most L1CAM Is not Associated with Extracellular Vesicles in Human Biofluids and iPSC–Derived Neurons
Source: Mol Neurobiol. 2025 Apr 10;62(8):10427–42. doi: 10.1007/s12035-025-04909-2 (PMC12289729; doi:10.1007/s12035-025-04909-2)
Supplement: Supplementary file 1 — Supplementary file1 (DOCX 4484 KB) [file 12035_2025_4909_MOESM1_ESM.docx]

**Supplementary figures**

**Fig. S1**


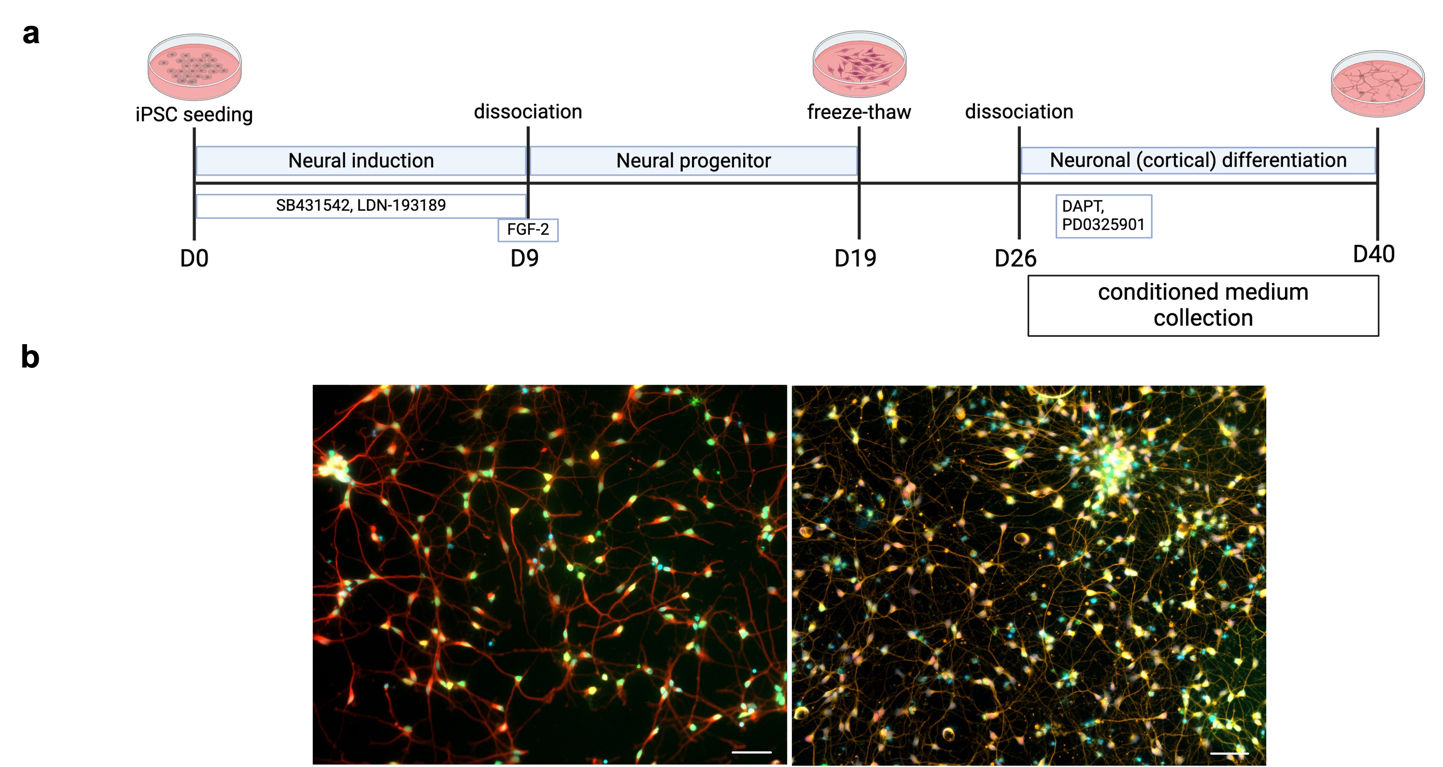


Fig. S1 **Immunocytochemistry (ICC) staining of human iPSC-derived cortical neurons shows presence of classical neuronal markers.**

**(a.)** Schematic diagram of the experimental protocol implemented for iPSC differentiation into iPSC-derived cortical neurons. Conditioned media for EV isolation was collected between DAI 27 and 40. **(b.)** Representative ICC images of iPSC-derived neurons stained for CTIP2 (layer V cortical neuron marker, in green), TBR1 (layer VI cortical neuron marker, in far-red), TUJ (neuronal differentiation marker, in red[left]/orange[right]) and DAPI (nuclear marker, in blue) at day 36 of neuronal differentiation. Magnification = 20X, scale bars = 50 μm

**Fig. S2**


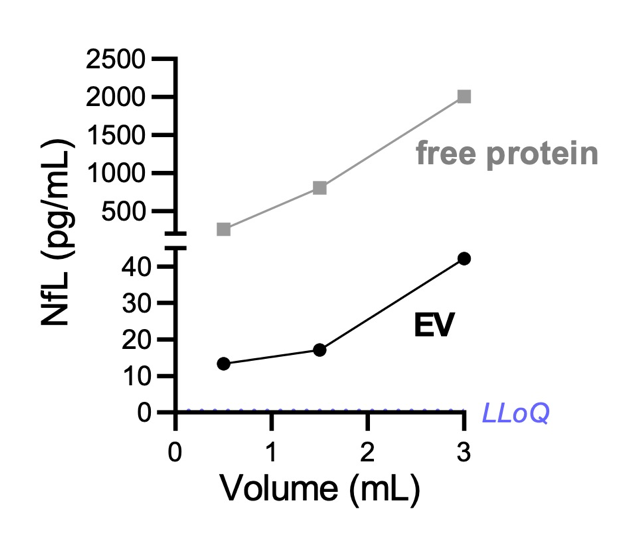


Fig. S2 **NfL levels in CSF-derived EVs isolated by PPT also increase with increasing CSF starting volume.**

The line plots denote SIMOA measurements of EV- associated (black) and free-floating (grey) NfL concentrations (pg/mL), with the purple dashed line represents the lower limit of quantitation (LLoQ) value (n=1)

**Tables**

**Table S1**


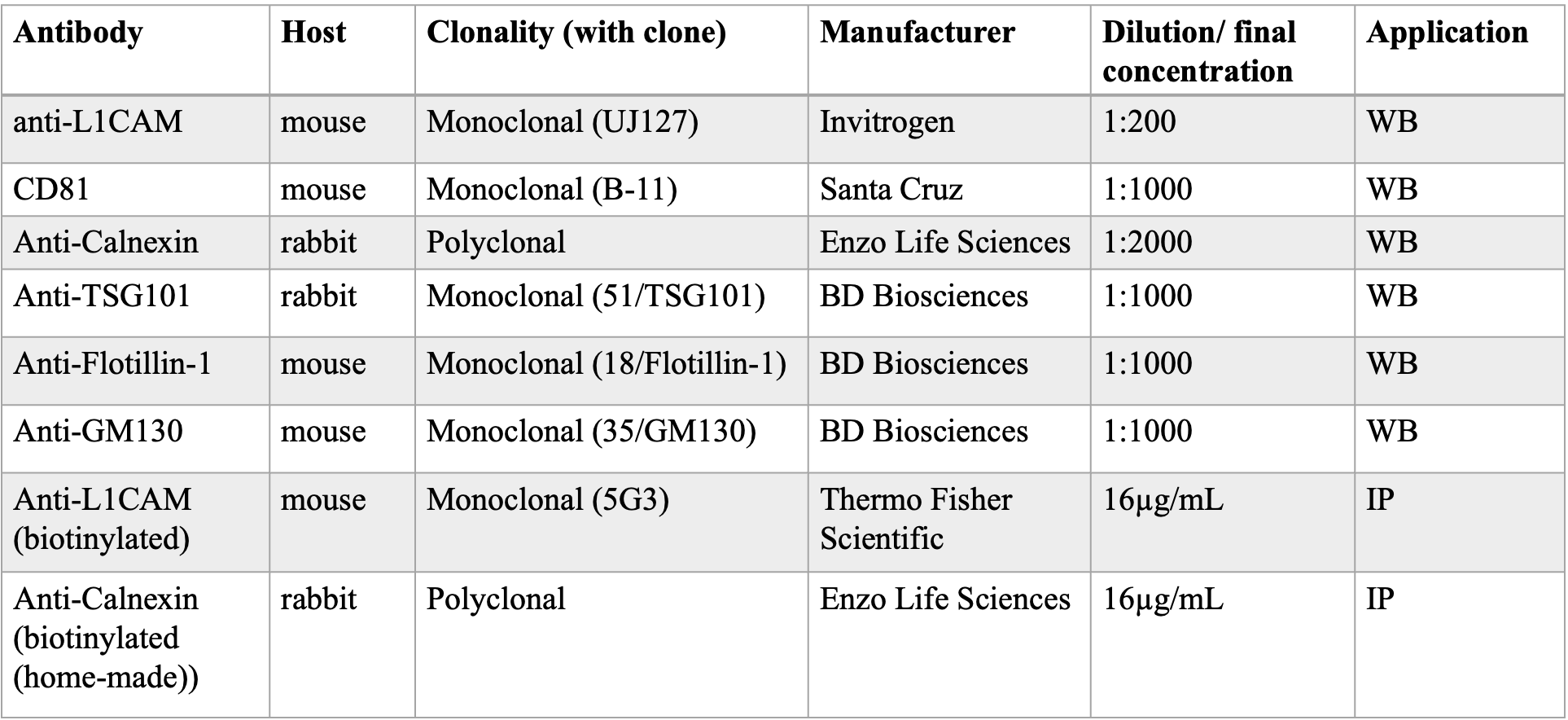


Table S1 **List of antibodies used.** *WB*, western blotting; *IP*, immunoprecipitation
